# P-536. Real-world Virologic and Safety Outcomes in People Living with HIV-1 Using Dolutegravir (DTG) + Lamivudine (3TC) in Asia Stratified by Viral Load (VL), Including in Individuals with High VLs

**DOI:** 10.1093/ofid/ofae631.735

**Published:** 2025-01-29

**Authors:** Andrés Doblado-Maldonado, Adrian Yit Reen Ooi, Jun Chen, Emilio Letang

**Affiliations:** ViiV Healthcare, Overijse, Vlaams-Brabant, Belgium; ViiV Healthcare, Overijse, Vlaams-Brabant, Belgium; Shanghai Public Health Clinical Center, Fudan University, Shanghai, Shanghai, China; ViiV Healthcare, Madrid, Spain, Madrid, Madrid, Spain

## Abstract

**Background:**

DTG + 3TC was effective for achieving virologic suppression (VS) in ART-naive adults living with HIV-1 with VL ≥ 100,000 c/mL in the GEMINI-1/-2 and STAT trials. Guidelines recommend DTG + 3TC as first-line ART in people with VL < 500,000 c/mL, but in real-world settings, people have initiated this regimen with higher VLs. To support treatment decisions, we summarize real-world evidence for DTG + 3TC in ART-naive people with high VL in Asia.

**Methods:**

Databases and congresses were searched (Jan 2013-Mar 2023) for non-interventional studies of DTG + 3TC use in N ≥ 10 people. Post hoc targeted searches were done to add recent (to Dec 2023) and non-English studies. Publications were screened for effectiveness/safety in ART-naive individuals in Asia, stratified by baseline (BL) VL. Results were summarized using lead study data by cohort to avoid overlap. High VL was ≥ or > 100,000 c/mL and very high VL ≥ or > 500,000 or 1,000,000 c/mL.

**Results:**

Of 283 publications, 21 were included, with ART-naive people using DTG + 3TC in China (n=551, 6 cohorts) and Türkiye (n=56, 1 cohort). Median age ranged from 37 to 66.5 years; 59% to 93% were male. When lead study n/N were pooled by VL, 149/262 (57%) people had high VL and 147/353 (42%) had very high VL (≥ 500,000 c/mL, 113/353; ≥ 1,000,000 c/mL, 40/152) at BL. VS (primarily VL < 50 c/mL) rates in lead studies in people with VL ≥ 100,000 vs < 100,000 c/mL were 52% vs 64% at Week (W)4 and 60% vs 93% at W12; corresponding rates in those with VL ≥ 500,000 vs < 500,000 c/mL were 5% vs 20% and 50% vs 75%. W48 VS rate was 92% for high VL (1 study; all had VL > 100,000 c/mL) and ranged from 64% to 96% for very high VL (vs 90%-92% with VL < 500,000 c/mL; 3 studies). In 1 study, 2 people with high VL had virologic rebound at W48 (resuppressed at W72). Outcomes were generally consistent between lead and non-lead within-cohort studies. One virologic failure was reported in a person with very high VL, due to non-adherence. Studies reported no discontinuations (n=1) or no discontinuations due to adverse events (AEs; n=7), drug-related AEs (n=1), or drug-related serious AEs (n=1). Few serious AEs occurred.

**Conclusion:**

Similar to clinical trials, real-world evidence in ART-naive individuals initiating DTG + 3TC in Asia demonstrated high VS rates and favorable safety outcomes, even when VL was high or very high at ART initiation.

**Disclosures:**

**Andrés Doblado-Maldonado, PhD**, GSK: Stocks/Bonds (Public Company)|ViiV Healthcare: Employee **Adrian Yit Reen Ooi, MBBS, MS**, GSK: Stocks/Bonds (Public Company)|ViiV Healthcare: Employee **Emilio Letang, MD, MPH, PhD**, GSK: Stocks/Bonds (Public Company)|ViiV Healthcare: Employee

